# Engineering isospectrality in multidimensional photonic systems: Multidimensional quasi-isospectrality in photonics

**DOI:** 10.1515/nanoph-2022-0740

**Published:** 2023-03-08

**Authors:** Dayeong Lee, Hyungchul Park, Sunkyu Yu

**Affiliations:** Intelligent Wave Systems Laboratory, Department of Electrical and Computer Engineering, Seoul National University, Seoul 08826, Korea

**Keywords:** coupled mode theory, coupled resonators, disordered system, isospectrality, perturbation, pseudo-inverse

## Abstract

Selective manipulation of energy levels plays an essential role in realizing multichannel wave devices. One of the representative examples is to utilize the concept of quasi-isospectrality: a family of wave systems with an almost identical spectrum except for a part of energy levels. Most approaches toward quasi-isospectrality have employed analytical methods based on symmetry or tridiagonalization, such as supersymmetry, Householder, or Lanczos transformations. Although such analytical approaches provide deterministic and stable designs based on operator factorizations, the mathematical strictness in the factorizations, at the same time, hinders isospectral engineering in a given multidimension. Here we develop the semi-analytical method for engineering isospectrality in multidimensional photonic systems. The method provides the systematic perturbation for the target energy level shifts by decomposing the allowed form of system changes into the perturbation basis. We demonstrate the isospectrality of lower-, higher-, and random-order states while imposing the designed shifts on the other states. The stability analysis shows that the accuracy of the method is determined by the ranges of isospectral state numbers and perturbation strength. The systematic, free-form, and multidimensional natures of the proposed method show great potential for the platform-transparent design of multichannel devices.

## 1 Introduction

Isospectrality [1]—the concept bridging different wave systems that provide an identical spectral response—is a representative example of the one-to-many relationships between waves and matter, which originate from the nonunique existence of the system potentials for a given physical observable. After the first suggestion of this concept with the famous question, “Can one hear the shape of a drum?” [2], and its answer [3] in acoustics, realizing a family of isospectral systems has attracted much attention in various fields of wave physics: photonics [4–16] and quantum physics [17–20]. For example, a given spectrum of resonant frequencies or propagating constants in photonic systems allows for multiple candidates of the configuration in complex-valued vectorial electromagnetic eigenmodes. Each configuration of the eigenmodes then leads to a different form of an optical potential—refractive index profiles or geometric structures—which supports an identical spectral response. Owing to the global phase matching condition between the evolutions of the eigenmodes in isospectral systems, achieving isospectrality is expected to play a critical role in multimodal functionalities, such as selective modal filtering [5, 7, 14, 21, 22]. In this application, the designed implementation of “quasi”-isospectrality—the isospectrality in the selective portion of a spectrum—is strongly desired to realize the independent handling of each mode, as demonstrated in the annihilation of the ground state in the unbroken supersymmetric transformation [5] and its application to mode demultiplexing [7].

Most of the previous approaches in realizing isospectrality have employed analytical methods based on system transformations, such as the Darboux transformation for supersymmetric optical structures [23] and the isospectrality between different dimensions using the Householder [14, 15] or Lanczos transformations [16]. Despite the great success of these analytical methods that guarantee the stable derivation of isospectral systems, the rigorous constraints of such methods, at the same time, prohibit the generality of the methods. For example, the basic form of supersymmetric transformations, which is based on the Darboux operator factorization [23], is applicable to one-dimensional (1D) systems governed by Schrödinger-form equations. The Householder [14, 15] and Lanczos [16] transformations are based on the matrix tridiagonalization, which is only applicable to the isospectrality between a higher-dimensional (two- (2D) or three-dimension (3D)) and a 1D system. Although many efforts have been developed to realize the multidimensional extension of supersymmetric transformations by using the separation of variables [8, 11], time-varying design [24], or Moutard transformation [25], these methods still undergo several restrictions on the possible forms of optical potentials or governing equations. For practical applications, the generalized method for isospectrality in a given multidimension is still a challenging issue, requiring the handling of multidimensional systems, general forms of governing equations, and free-form selection of eigenmodes for quasi-isospectrality. Alleviating the mathematical constraints in system transformations may provide a clue for this issue, for example, by using semi-analytical approaches.

In this paper, we propose the semi-analytical approach to quasi-isospectrality in the regime of weak perturbation. For a general Hamiltonian equation, we analyze the relationship between system parameters and energy level shifts using the first-order perturbation theory, which provides the condition of the system perturbation for the target form of quasi-isospectrality. We employ this method to 2D deformed optical lattices, achieving the significant suppression of the energy shift for the target eigenmode. We analyze the effect of the number of target eigenmodes and the strength of perturbations on the validity of the method. The multimodal filtering in 2D systems is also demonstrated as an application example. The proposed approach is platform-transparent, systematic, and free-form in selecting the range of isospectrality, which will enable more practical forms of isospectral transformations.

## 2 Quasi-isospectral perturbation

Perturbation theory allows for analyzing the effect of relatively small changes on wave behaviors by employing known solutions. We utilize this theory in designing quasi-isospectral optical potentials: the potentials possessing almost the same eigenspectra except for a set of selected ones. This approach can be considered the semi-analytical generalization of supersymmetry transformation into multidimensional problems.

**Figure 1:**
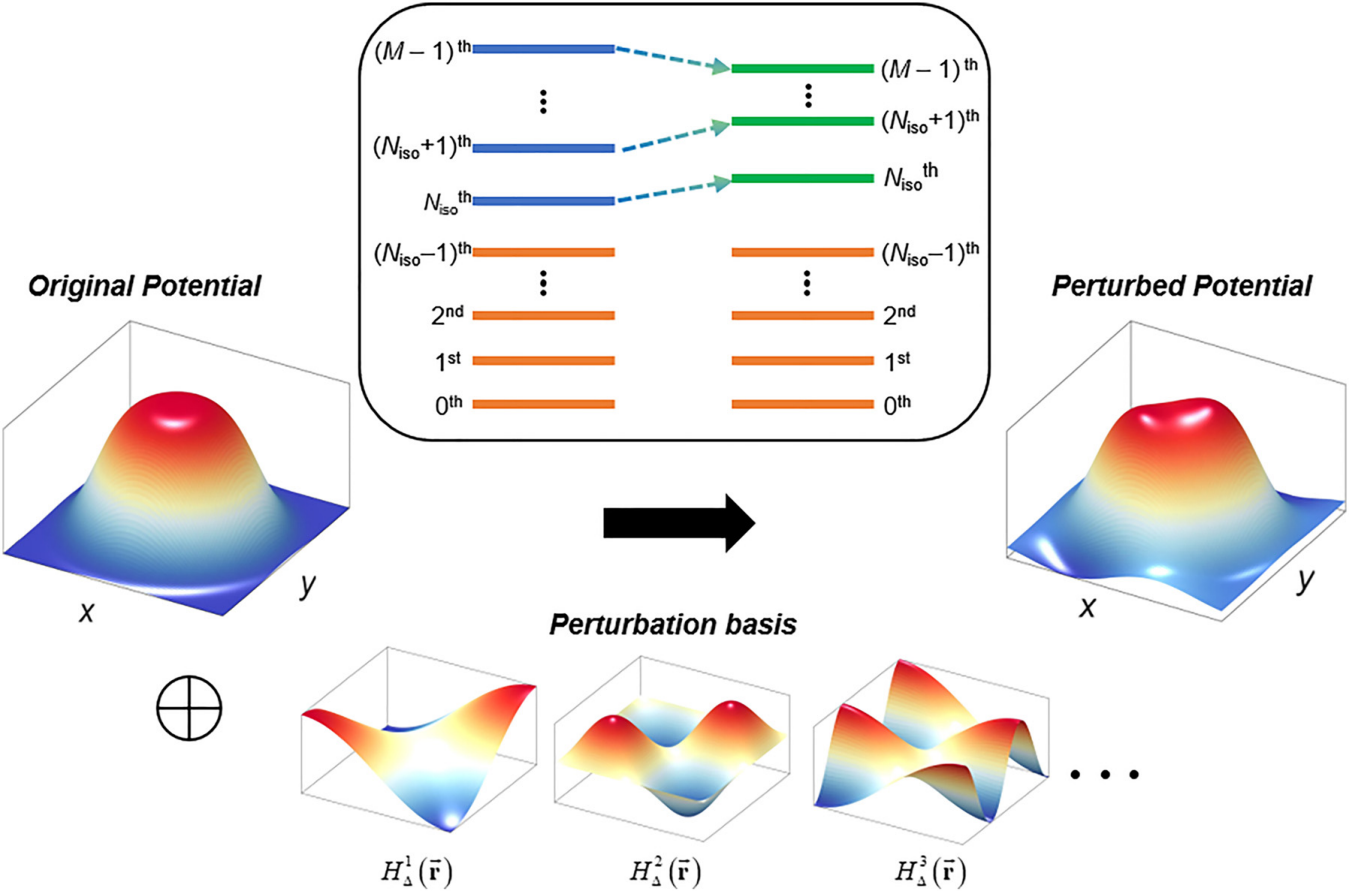
Quasi-isospectrality using perturbation theory. A schematic for quasi-isospectral transformation of a potential using perturbation theory. The goal of the method is to preserve the energy levels of the 0th ∼ (*N*_iso_ – 1)th modes, while shifting the remaining modes (*N*_iso_th ∼ (*M* – 1)th modes), where *N*_iso_ denotes the element number of *Q*. The perturbation is decomposed into the perturbation basis {*H*_Δ_^*n*^} to achieve the analytical relation between the designed energy level shifts and the magnitudes of each perturbation component.

Suppose the Hamiltonian eigenvalue equation *H*_0_|*ψ*_*m*_^0^> = *E*_*m*_^0^|*ψ*_*m*_^0^> without any restrictions on the dimensionality and platform of the system of interest except for the system Hermiticity and static condition. We mainly focus on achieving quasi-isospectrality in *M* number of bound states (*m* = 0, 1, …, *M* – 1, Figure 1), while assuming nondegeneracy for simplicity. According to the nondegenerate perturbation theory [26], when the perturbed Hamiltonian *H* = *H*_0_ + *H*_Δ_ leads to *H*|*ψ*_*m*_> = *E*_*m*_|*ψ*_*m*_>, the first-order perturbation of the eigenvalue *E*_*m*_ is

(1) $$\begin{array}{ll} {E_{m}}^{1} & ≜E_{m}-{E_{m}}^{0}\\ & =\left\langle {\psi_{m}}^{0}\right|H_{\Delta }\left|{\psi_{m}}^{0}\right\rangle .\;\left(m=0,1,\ldots ,M-1\right) \end{array}$$

The desired condition of quasi-isospectrality for demultiplexing applications, for example, the selective phase matching condition for the finite number of states, is to satisfy the following condition:

(2) $$\begin{array}{c} {E_{m}}^{1}=0,\left(m\in Q\right)\\ \neq 0,\left(m\notin Q\right) \end{array}$$

where *Q* is a set of the selected integers between 0 and *M* – 1. For example, the isospectrality, except for the ground and first excited states, is defined with *Q* = {2, 3, …, *M* – 1}. When *m* ∉ *Q*, the value of *E*_*m*_^1^ can be designed.

To introduce the inverse design of the perturbation under Eq. (2), we assume the decomposition of the Hamiltonian perturbation into *N* number of the perturbation “basis” *H*_Δ_^*n*^ (*n* = 0, 1, …, *N* – 1), which can be a specific structural or material change in the system (Figure 1). As shown in the basis representation, a set of *H*_Δ_^*n*^ is assumed to be mutually independent and to be defined in the same Hilbert space with *H*_0_. When the magnitude of each basis perturbation is *h*_*n*_, the Hamiltonian perturbation is decomposed as follows:

(3) $$H_{\Delta }=\overset{N-1}{\underset{n=0}{\sum }}h_{n}{H_{\Delta }}^{n}.$$

In Eq. (3), the set {*h*_*n*_} then corresponds to the system parameters, such as material distributions or geometric structures, fully describing the system perturbation. Our perturbation approach aims to find {*h*_*n*_} for a given set of {*E*_*m*_^1^} that describes the quasi-isospectral condition.

Equations (1) and (3) lead to the following *M* × *N* matrix equation:

(4) $$\begin{array}{ll} & \left[\begin{array}{cccc} \left\langle {\psi_{0}}^{0}\right|{H_{\Delta }}^{0}\left|{\psi_{0}}^{0}\right\rangle & \left\langle {\psi_{0}}^{0}\right|{H_{\Delta }}^{1}\left|{\psi_{0}}^{0}\right\rangle & \ldots & \left\langle {\psi_{0}}^{0}\right|{H_{\Delta }}^{N-1}\left|{\psi_{0}}^{0}\right\rangle \\ \left\langle {\psi_{1}}^{0}\right|{H_{\Delta }}^{0}\left|{\psi_{1}}^{0}\right\rangle & \left\langle {\psi_{1}}^{0}\right|{H_{\Delta }}^{1}\left|{\psi_{1}}^{0}\right\rangle & \ldots & \left\langle {\psi_{1}}^{0}\right|{H_{\Delta }}^{N-1}\left|{\psi_{1}}^{0}\right\rangle \\ \vdots & \vdots & \ddots & \vdots \\ \left\langle {\psi_{M-1}}^{0}\right|{H_{\Delta }}^{0}\left|{\psi_{M-1}}^{0}\right\rangle & \left\langle {\psi_{M-1}}^{0}\right|{H_{\Delta }}^{1}\left|{\psi_{M-1}}^{0}\right\rangle & \ldots & \left\langle {\psi_{M-1}}^{0}\right|{H_{\Delta }}^{N-1}\left|{\psi_{M-1}}^{0}\right\rangle \end{array}\right]\left[\begin{array}{c} h_{0}\\ h_{1}\\ \vdots \\ h_{N-1} \end{array}\right]=\left[\begin{array}{c} {E_{0}}^{1}\\ {E_{1}}^{1}\\ \vdots \\ {E_{M-1}}^{1} \end{array}\right]. \end{array}$$

When *M* ≠ *N*, the solution of Eq. (4) may not exist or be non-unique. By introducing the generalized (or pseudo-) inverse [27], such as the Moore–Penrose pseudo-inverse [28], we can derive the best fit of {*h*_*n*_} for the quasi-isospectral eigenvalues {*E*_*m*_^1^} defined by *Q*, the original Hamiltonian *H*_0_, and the setting of the perturbation basis *H*_Δ_^*n*^.

## 3 Quasi-isospectral coupled systems

The general form of Eq. (4) enables the inverse design of quasi-isospectrality in any dimensions and platforms when the system can be described by the eigenvalue problem without the degeneracy. As an example, we apply the method to deformed optical lattices, which can be composed of optical resonators [29], waveguides [30], or metamaterials [31]. Due to the deformation, we can maintain the non-degeneracy assumption, while the generalization to degenerate systems is straightforward using degenerate perturbation theory [26].

### 3.1 Perturbation model of coupled resonator lattices

**Figure 2:**
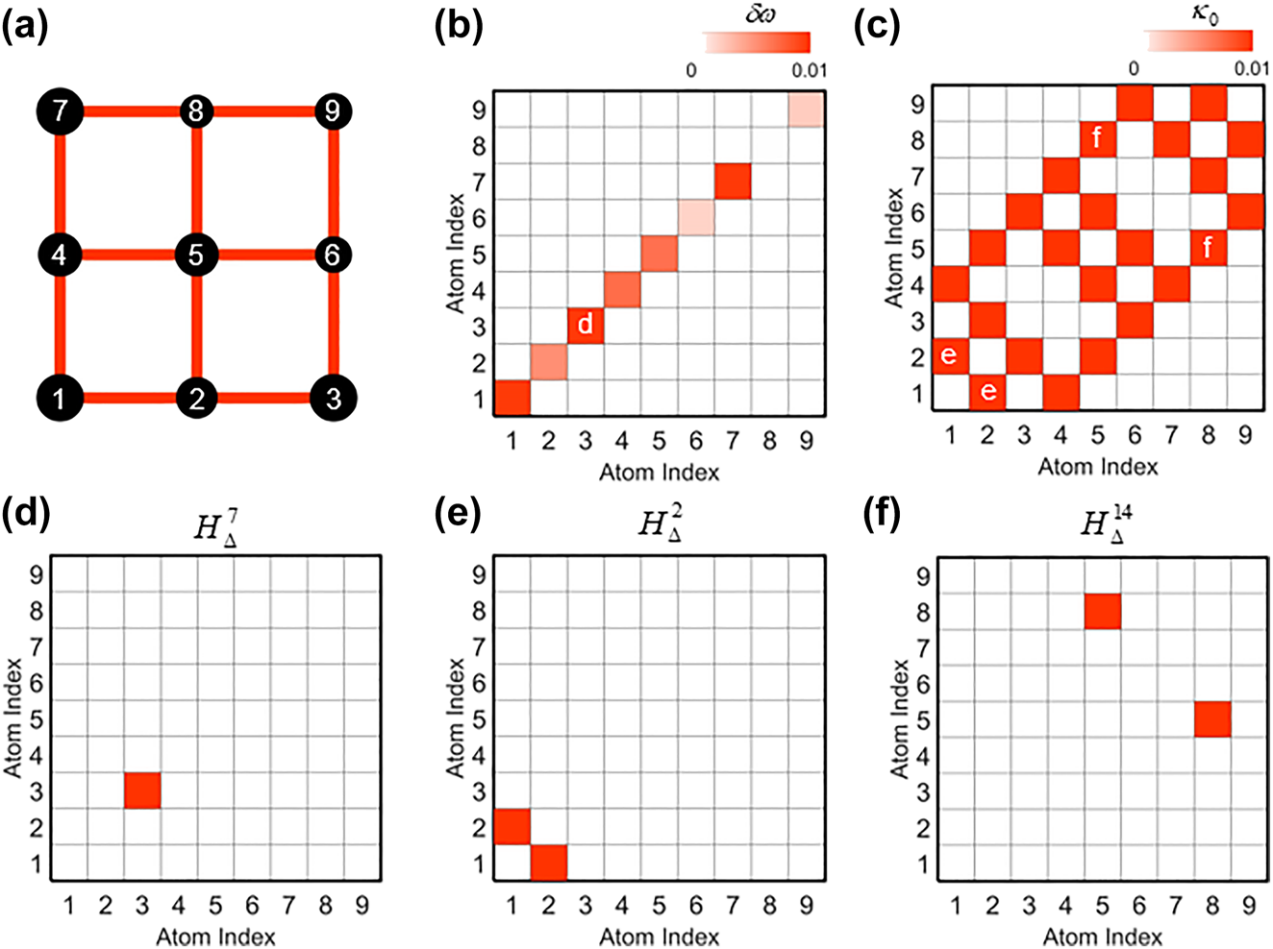
Perturbative engineering of coupled resonator lattices. (a) A schematic of an unperturbed initial lattice with disordered resonant frequencies. Irregular radii of atoms denote the degree of disorder. (b, c) Examples of (b) diagonal (i.e., the detuning of resonant frequencies *δω* = *ω*_*k*,0_ – *ω*_L_) and (c) off-diagonal (i.e., coupling coefficients *κ*_0_) components of *H*_0_. The lowest resonant frequency *ω*_L_ is set to be 1. (d–f) Examples of the Hamiltonian perturbation basis: (d) *H*_Δ_^7^, (e) *H*_Δ_^2^, and (f) *H*_Δ_^14^. Perturbations can be either (d) diagonal or (e, f) off-diagonal. The symbols d, e, and f in (b, c) denote the perturbation basis in (d–f).

Without loss of generality, we assume the 2D *L*_*x*_ × *L*_*y*_ square lattice composed of weakly coupled optical resonators (Figure 2a for *L*_*x*_ = *L*_*y*_ = 3). Such an *M*-resonator (*M* = *L*_*x*_ × *L*_*y*_) system can be described by the temporal coupled mode theory (TCMT) [29, 32] as follows:

(5) $$i\frac{\partial }{\partial t}\psi =-\left(\Omega +K\right)\psi ,$$

where *ψ* = [*φ*_1_, *φ*_2_, …, *φ*_*M*_]^T^ is the coupled mode state vector defined with the field amplitude *φ*_*k*_ inside the *k*th resonator, and Ω and *K* are the on-site and hopping matrices, respectively. The on-site matrix Ω is diagonal, and its *k*th diagonal element *ω*_*k*_ denotes the resonant frequency of the *k*th resonator. The hopping matrix *K* describes the coupling network of the system, where its (*p*, *q*) element *K*_*pq*_ denotes the coupling coefficient between the *p*th and *q*th resonators. Equation (5) can be re-expressed as the eigenvalue problem *H*_0_ *ψ*_*m*_^0^ = *E*_*m*_^0^ *ψ*_*m*_^0^ with the Hamiltonian matrix *H*_0_ = –(Ω + *K*) (Figure 2b for the Hamiltonian of Figure 2a). The eigenfrequency of the coupled mode *ψ*_*m*_^0^ is then obtained as *ω*_*m*_^0^ = –*E*_*m*_^0^. To leave out the degeneracy in the system, we set the initial resonant frequency as *ω*_*k*_ = *ω*_*k*,0_ = *ω*_L_ + *u*(0, *δ*), where *ω*_L_ is the lowest resonant frequency, and *u*(*a*, *b*) represents the uniform random distribution between the values *a* and *b*. For the free-form inverse design, we assume the inter-elemental coupling through the intermediate zero-field optical elements [33, 34], which allows for the wide-range manipulation of the coupling coefficient in its sign [35] and magnitude [36]. The initial coupling coefficient is set to be *K*_*pq*_ = *κ*_0_, when *p* and *q* depict the nearest-neighbor elements.

The proposed system then supports the *M* (= *L*_*x*_ × *L*_*y*_) number of bound states. Due to the reciprocity *K*_*pq*_ = *K*_*qp*_, the 2D square-lattice system supports 3*L*_*x*_ *L*_*y*_ – *L*_*x*_ – *L*_*y*_ degrees of freedom for the system parameters: coupling coefficients and resonant frequencies. We exploit full design freedom for the number of perturbation basis vectors *N* = 3*L*_*x*_ *L*_*y*_ – *L*_*x*_ – *L*_*y*_. Figure 2d–f shows three examples of *H*_Δ_^*n*^ defined by some system parameters in Figure 2a, which operate as the basis of the highlighted elements of *H*_0_ in Figure 2b and c. By employing the basis perturbations *H*_Δ_^*n*^, the initial eigenmodes *ψ*_*m*_^0^, and a given eigenvalue shift {*E*_*m*_^1^}, we deterministically achieve the magnitudes of each perturbation component {*h*_*n*_} using Eq. (4) and the Moore–Penrose pseudo-inverse [28].

### 3.2 Quasi-isospectral perturbation for selective states

In examining the proposed perturbation model for coupled resonator lattices, we define the number of conserved eigenfrequencies (or the size of *Q*) as *N*_iso_. Among numerous candidates for selecting *N*_iso_ from *M* nondegenerate eigenmodes, we focus on achieving isospectrality for continued lower-order (or smaller *m*) eigenmodes, higher-order eigenmodes (or larger *m*), and randomly distributed order of eigenmodes. Although the other eigenmodes can experience the designed shifts, we introduce the uniformly random shift *E*_*m*_^1^ = *u*(0, *A*) for *m* ∉ *Q* without any loss of generality.

**Figure 3:**
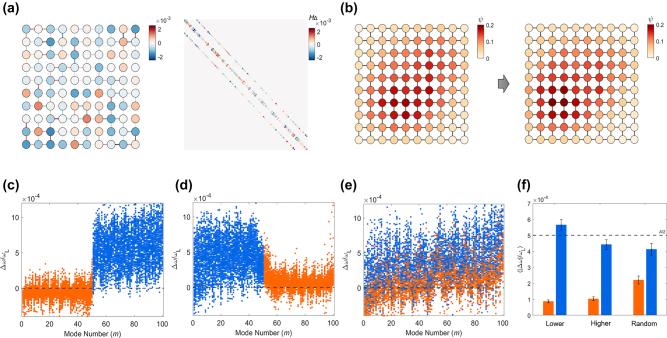
Quasi-isospectral engineering in coupled resonator lattices. (a) An example of the deformed optical lattice (left) and the corresponding *H*_Δ_ matrix (right). Colors in the left figure represent the changes in the system parameters *ω*_*k*_ and *K*_*pq*_, which correspond to *H*_Δ_ in the right figure. (b) The ground state field distributions before and after the perturbation for the case of (a). (c–e) Eigenfrequency shifts for the 100 realizations of (c) lower-, (d) higher-, and (e) random-order eigenmodes. The orange dots represent the target eigenmodes of quasi-isospectrality, and the blue dots represent the remaining modes. (f) The ensemble average of |Δ*ω*|/*ω*_L_ for each case of (c–e) (black dashed line for *A*/2). Each error bar denotes a quarter of the standard deviation for an ensemble. *L*_*x*_ = 10, *L*_*y*_ = 10, *M* = 100, *N* = 280*, ω*_L_ = 1, *δ* = 0.01, *κ*_0_ = 0.01*, N*_iso_ = 50, and *A* = 0.001 for all cases.

Figure 3a–c shows the isospectrality in lower-order eigenmodes. For an example of the Hamiltonian perturbation *H*_Δ_ in Figure 3a, the resulting change of the ground states is shown in Figure 3b. As shown in Figure 3c, which describes the eigenfrequency shift Δ*ω*_*m*_ for the 100 realizations of a random ensemble with the random variables *ω*_*k*,0_ and *E*_*m*_^1^, the proposed method successfully allows for quasi-isospectrality for the target eigenmodes (orange dots), while assigning the uniformly random perturbation to the remaining eigenmodes (blue dots).

The comparison of the isospectral designs for lower- (Figure 3c), higher- (Figure 3d), and random- (Figure 3e) order eigenmodes exhibits interesting features in the validity of the methods. When comparing Figure 3c and d, achieving the isospectrality of lower-order eigenmodes that support the slower spatial oscillation provides higher accuracy. In a similar context, the collective realization of the isospectrality for the nearby eigenmodes, which support the similar degrees of spatial oscillations, also provides higher accuracy than random order cases (Figure 3c and d vs. Figure 3e). These results demonstrate that the validity of achieving quasi-isospectrality, which will be determined by the magnitude of higher-order perturbations, is strongly related to the spatial resolution of eigenmodes.

To verify this observation in a statistical manner, we introduce the ensemble average shift ⟨|Δ*ω*|/*ω*_L_⟩ where ⟨…⟩ denotes the ensemble average for 100 realizations. We separately define the eigenfrequency shift for each realization, as |Δ*ω*(*Q*)| = Σ_*m*∈*Q*_|Δ*ω*_*m*_|/*N*_iso_ (orange in Figure 3f) and |Δ*ω*(*Q* ^*c*^)| = Σ_*m*∉*Q*_|Δ*ω*_*m*_|/(*M* – *N*_iso_) (blue in Figure 3f). The observations in Figure 3c–e are well-confirmed with Figure 3f, while the setting of isospectrality for nearby eigenmodes is more critical to the accuracy of the method.

### 3.3 Stability of quasi-isospectral perturbation

To further examine the stability of the quasi-isospectral perturbation, we investigate the validity of the method for different numbers of isospectral modes (*N*_iso_) and the average frequency shift of the remaining modes (*A*). To quantify the error of the design, we define the relative shift of the target eigenmodes as

(6) $$\sigma \equiv \frac{\left\langle \left|\Delta \omega \left(Q\right)\right|\right\rangle }{\left\langle \left|\Delta \omega \left(Q^{c}\right)\right|\right\rangle }.$$

**Figure 4:**
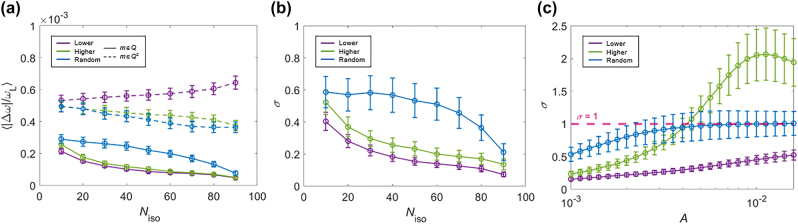
Stability analysis. (a) The relationship between ⟨|Δ*ω*|/*ω*_L_⟩ and *N*_iso_ for lower-, higher-, and random-order cases. The solid lines denote the target eigenmodes for isospectrality, and the dashed lines denote the remaining eigenmodes. (b) The variation of the error *σ* with respect to *N*_iso_ for lower-, higher-, and random-order cases. In (a, b), *A* = 0.001. (c) The variation of *σ* with respect to *A* for lower-, higher-, and random-order cases (red dashed line for *σ* = 1). In (c), *N*_iso_ = 50. For (a–c), 100 realizations are analyzed for each data point. The error bar denotes a quarter of the standard deviation for an ensemble. All the other parameters are the same as those in Figure 3.

Figure 4a and b show the dependency of the eigenfrequency shifts |Δ*ω*| and the following errors *σ* for the target and remaining eigenmodes. While the trend of Figure 3 is maintained, the increase of *N*_iso_, which results in more collective conservation of the original eigenfrequencies, provides better accuracy, as shown in the decrease of *σ* for all cases.

Another important parameter for determining the validity of the method is the magnitude of the target eigenfrequency shift *A* of the remaining eigenmodes. Due to Eq. (4), the parameter *A* directly corresponds to the perturbation strength, consequently quantifying the validity of the first-order perturbation. As shown in Figure 4c, the increase of *A* naturally derives the degradation of the accuracy with increasing *σ*. In the same context with Figures 3 and 4a and b, the quasi-isospectrality for lower-order modes is the most stable. The validity of the proposed method, which should be quantified with the condition *σ* < 1 for the relatively larger suppression of |Δ*ω*| in the target eigenmodes, is maintained in the range of *A* < ∼0.004 (green line in Figure 4c).

It is also worth mentioning that the accuracy of the proposed method depends on the relative size of the perturbation basis *N* with respect to the number of the eigenmodes of interest *M*. The relationship between *N* and the accuracy of the isospectral engineering is examined with various metrics in Supplementary Note S1: the averaged frequency shift of the entire spectrum |Δ*ω*|/*ω*_L_, the metric quantifying the isospectral condition *σ*, and the mean squared error from the target shift. The result exhibits the tradeoff between the feasibility of the practical implementation with respect to *N* and the accuracy of quasi-isospectral engineering.

In Supplementary Note S2, we also investigate the upper bound of the difference between the spectral shifts in *Q* and *Q* ^*c*^, which is a critical parameter for the independent manipulation of the target eigenmodes from the remaining ones: multimodal filtering in multidimensions. The analysis shows that the upper bound is determined by the acceptable range of the first-order perturbation theory, showing ∼30% shift of the initial eigenfrequency obtained with small *N*_iso_ and large *A* in the lower-order case.

### 3.4 Multimodal filtering in two-dimensional systems

As an application example of quasi-isospectral engineering in multidimensional systems, we demonstrate two-dimensional modal filtering: the filtering of *Q* from the entire eigenmodes. Compared with conventional supersymmetry transformations that allow for the filtering of a higher set of eigenmodes in 1D systems [10, 21], our method enables the filtering of eigenmodes with an arbitrary order in multidimensions.

**Figure 5:**
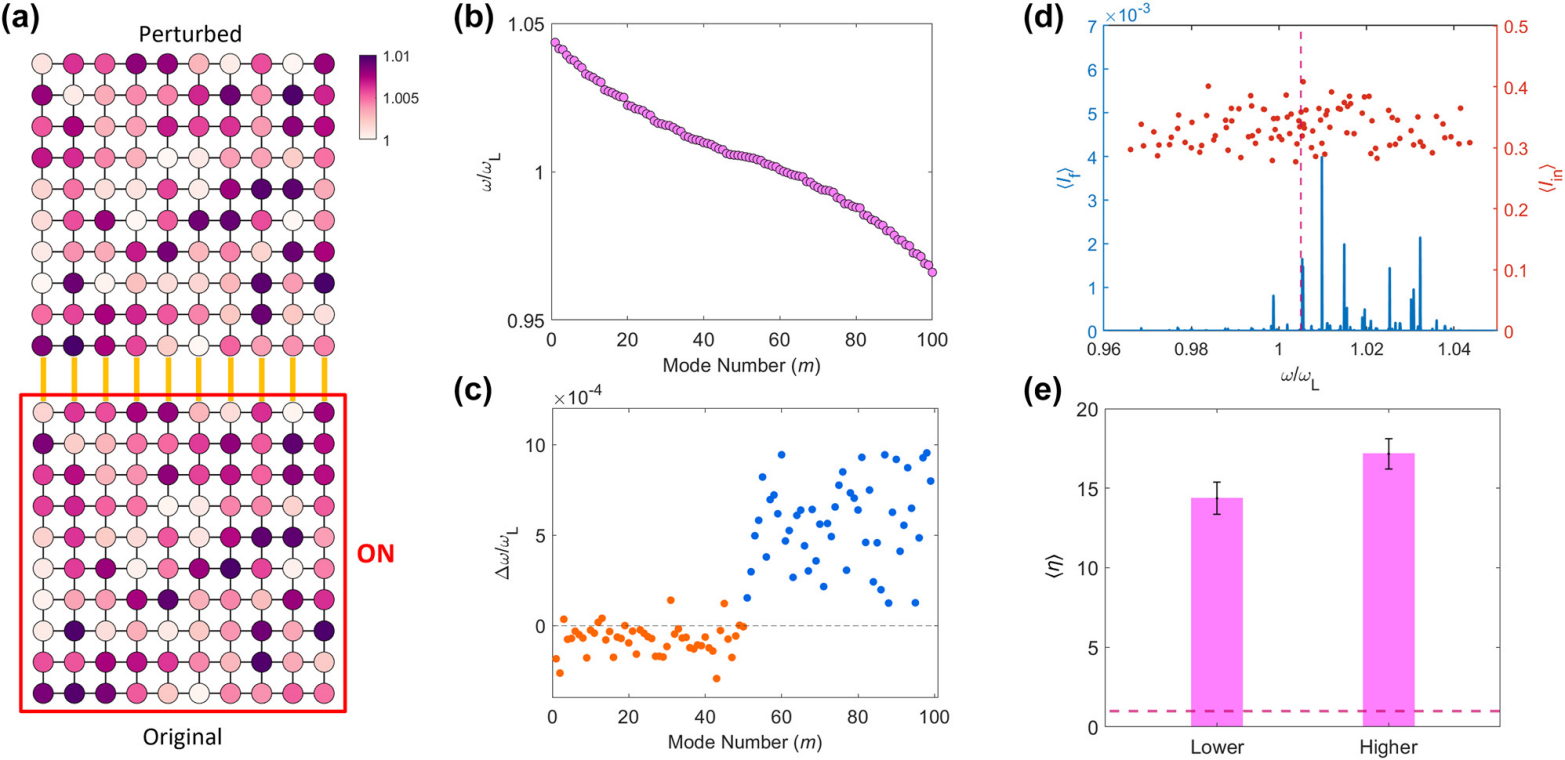
Multimodal filtering in 2D lattices. (a) The schematic of an example coupled lattice composed of the original lattice (bottom) and the designed quasi-isospectral lattice (top). The color in each resonator denotes the resonant frequency. Yellow solid lines between the boundary resonators of the lattices represent the uniform external coupling coefficient *κ* = *κ*_0_/100 = 10^−4^. The red box around the original lattice denotes the region where the initial Dirac delta excitation is applied. (b) Eigenfrequencies of the original lattice in (a). (c) Eigenfrequency shifts from the eigenspectrum in (b) obtained with the quasi-isospectral engineering. (d) The spectral intensity averaged over the resonators of the perturbed lattice during the time *T* (⟨*I*_f_⟩, blue lines with the left axis), and the input intensity of each eigenmode on the original lattice (⟨*I*_in_⟩, red dots with the right axis). The dashed line in (d) denotes the boundary between *Q* and *Q* ^*c*^. (e) The averaged *η* with a set of random inputs for lower- and higher-order mode cases. The dashed line in (e) denotes ⟨*η*⟩ = 1. 100 random incidences are analyzed for the average. The error bar denotes the quarter of the standard deviation for an ensemble. All the other parameters are the same as those in Figure 3.

We investigate the coupled system consisting of the original lattice weakly coupled with the perturbed lattice designed with Eq. (4) (Figure 5a). The lattices are coupled with the external coupling coefficient *κ* between the nearby boundary resonators. Two lattices share the same eigenspectra for *m* ∈ *Q* in Figure 5b and c.

We set an initial field in the original lattice as the temporal Dirac delta function at the time *t* = 0 with the spatial superposition of the entire eigenmodes of the original lattice. The amplitude of each eigenmode in the input is randomly weighted with the complex-valued amplitude having the magnitude *u*(0, 1) and the phase 2*πu*(0, 1), leading to the statistically uniform ensemble-averaged intensity for each eigenmode (red dots on Figure 5d). In this environment, the spectral intensity in the perturbed lattice during the time *T* = 5 × 10^4^(2*π*/*ω*_L_) is mainly distributed on a high frequency regime of *Q*, which demonstrates the desired multimodal filtering function (blue solid lines in Figure 5d).

To quantify the filtering efficiency for a realization for a given input, we define the following parameter:

(7) $$\eta \equiv \bar{I_{\text{f}}\left(Q\right)}/\bar{I_{\text{f}}\left(Q^{c}\right)},$$

where *I*_f_(*S*) are the filtered spectral intensities averaged over the perturbed lattice for the spectral set *S* (= *Q* or *Q* ^*c*^), and the upper bar denotes the spectral average over *S*.

When we calculate the averaged efficiency ⟨*η*⟩ for a set of 100 random inputs, the filtered signal intensity in *Q* is much larger than that in *Q* ^*c*^ with the efficiencies of about 11.58 dB for the lower-order (or high-frequency filtering) case and 12.36 dB for the higher-order (or low-frequency filtering) case (Figure 5e). The origins of the unwanted operations—the couplings in the sidebands of *Q* or in the eigenmodes of *Q* ^*c*^—include the spectral broadening from nonnegligible *κ* and the accidental phase matching or the limit of the first-order perturbation theory determined by the perturbation strength *A*. In Supplementary Note S3, we examine the filtering efficiency more in detail with varying *κ* and *A*.

## 4 Conclusions

We developed the semi-analytical design method for quasi-isospectral optical platforms using the perturbation theory. Because the method is developed with the general Hamiltonian equation, the proposed theoretical method is platform-transparent. As an example, we studied the 2D weakly coupled optical lattices for the free-form realization of quasi-isospectrality: the conservation of the resonances for lower orders, higher orders, and randomly-selected orders. The analysis for the stability of the method, including the modal range of the isospectrality and perturbation strength, demonstrates the valid range of the method. As an example of our engineering technique, we demonstrated multimodal wave filtering in 2D systems.

Although we treated discrete cases, the generality of our formulation allows for handling continuous systems: of the quasi-isospectral engineering in continuous material profiles such as permittivity. In such a problem, any forms of an orthogonal basis that spans a given space can be employed to be the perturbation basis. For example, when the governing equation is [∇^2^ + *V*(**r**)]*ψ*_*m*_ = *E*_*m*_ *ψ*_*m*_ where *V*(**r**) is the optical potential, and *ψ*_*m*_ and *E*_*m*_ are eigenmodes and eigenvalues, respectively, we can develop a basis {*φ*} for expanding the optical potential by solving the Laplace equation ∇^2^ *φ* = *O* with a given boundary condition (e.g., Dirichlet or Neumann boundary conditions).

Due to the platform transparency and multidimensional generality, the method can extend the design freedom in achieving the functionalities based on quasi-isospectrality, such as the multimode control in lasing [21, 22] or engineering scattering or bandgap design in disordered systems [11, 13, 37, 38, 39, 40]. For more practical implementation, the method needs to be generalized with higher-order perturbation terms for better accuracy and degenerate perturbation theory for symmetry-protected multidimensional systems. For example, the extension of our method up to second-order perturbations, which results in multivariate quadratic equations containing the second-order terms [26], will require numerical assessments to obtain the coefficient for each perturbation basis vector. It is also worth mentioning that non-Hermitian perturbation theory [41] will enable the generalization of the proposed method to the systems possessing time-varying parameters [42], gain or loss materials [43], and coupling to the external environment.

## Supplementary Material

Supplementary Material Details
