# Supplementary material for: Engineering isospectrality in multidimensional photonic systems: Multidimensional quasi-isospectrality in photonics
Source: Nanophotonics. 2023 Mar 8;12(13):2593–601. doi: 10.1515/nanoph-2022-0740 (PMC11501229; doi:10.1515/nanoph-2022-0740)
Supplement: Supplementary file 1 — Supplementary Material Details [file j_nanoph-2022-0740_suppl.pdf]

Supplementary Information for "Engineering isospectrality in multidimensional photonic systems"

Dayeong Lee, Hyungchul Park, and Sunkyu Yu[*]

Intelligent Wave Systems Laboratory, Department of Electrical and Computer Engineering, Seoul National University, Seoul 08826, Korea

E-mail address for correspondence: [*]sunkyu.yu@snu.ac.kr

Note S1. The effect of a perturbation basis on accuracy

Note S2. The upper bound of Q^c perturbation

Note S3. Efficiency of multimodal filtering

Note S1. The effect of a perturbation basis on accuracy

In the proposed method, the Hamiltonian perturbation is decomposed into N number of perturbation basis vectors. In the main text, we set N to express full degrees of freedom of the system parameters, that is, $N = 3L_xL_y - L_x - L_y$ for the $L_x \times L_y$ square lattice system: L_xL_y resonance frequencies and $2L_xL_y - L_x - L_y$ coupling coefficients. In this note, we investigate the effect of decreasing N to examine the relationship between the accuracy of the method and the number of perturbation basis vectors. To quantify the error of the design, we introduce the mean squared error to measure the difference between the target shifts $\Delta\omega_{m,\mathrm{target}}$ and the achieved shifts $\Delta\omega_{m,\mathrm{actual}}$ of the eigenmodes with $\{\Delta\omega_m\}$, as follows:

$$L_2 \equiv \frac{1}{\omega_\mathrm{L}} \sqrt{\frac{1}{M} \sum_{m=0}^{M-1} (\Delta\omega_{m,\mathrm{target}} - \Delta\omega_{m,\mathrm{actual}})^2}, \quad \text{(S1)}$$

where M is the total number of eigenmodes.

Figure S1 shows the ensemble-averaged $|\Delta\omega|/\omega_\mathrm{L}$ (Fig. S1a), the relative shift of the target (Q) eigenmodes σ (Fig. S1b), and the ensemble-averaged mean squared error L_2 (Fig. S1c) obtained with a given realization having the fixed target spectrum. In Fig. S1a, the ensemble averaged $|\Delta\omega(Q^c)|/\omega_\mathrm{L}$ increases to $A/2$ with larger N (pink dashed line), which matches with the expectation value of the target shift $E_m^1 = u(0, A)$ for $m \notin Q$. As shown in Fig. S1b, σ decrease with larger N, exhibiting better isospectrality for the target eigenmodes in Q according to Eq. (6). The error of the design L_2 also decreases with larger N (Fig. S1c), demonstrating that increasing N guarantees the accuracy of our method due to the extended design space and the following extension of the state space for isospectral engineering. Notably, despite the increase of accuracy with larger N, there is a trade-off relationship in terms of the practical realization; larger N requires manipulating a larger number of system parameters in discrete systems and spatially more sensitive control of optical potentials in continuous systems.

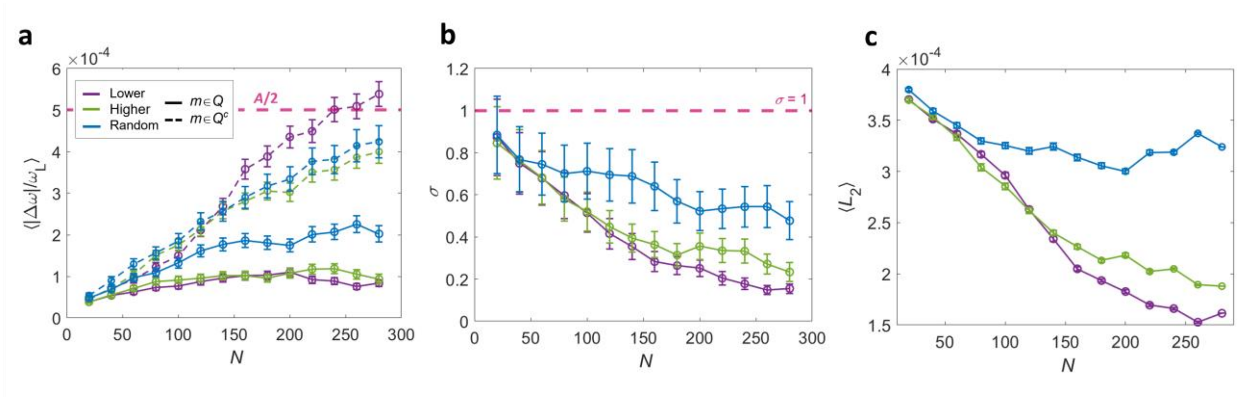

Figure S1. The effect of a perturbation basis. (a) The ensemble-averaged $|\Delta\omega|/\omega_L$ in the range of $N \in (0, 3L_xL_y - L_x - L_y]$ for lower-, higher-, and random-order cases. The solid lines correspond to the target eigenmodes (Q) for isospectrality, and the dashed lines correspond to the remaining eigenmodes (Q^c). (b) The relative shift of the target eigenmodes σ in the range of $N \in (0, 3L_xL_y - L_x - L_y]$. The red dashed line denotes $\sigma = 1$. (c) The ensemble-averaged mean squared error L_2 in the range of $N \in (0, 3L_xL_y - L_x - L_y]$. In each ensemble, 5×10^3 random selections of basis vectors are examined for the fixed target spectrum. $L_x = 10$, $L_y = 10$, $M = 100$, $\omega_L = 1$, $\delta = 0.01$, $\kappa_0 = 0.01$, $N_{iso} = 50$, and $A = 0.001$ for all cases.

Note S2. The upper bound of Q^c perturbation

We define the parameter δ_d to measure the difference between the spectral shifts of the isospectral set (Q) and the perturbed set (Q^c), as follows:

$$\delta_d \equiv \left\langle \frac{1}{\omega_L}\left(\overline{\Delta\omega(Q^c)} - \overline{\Delta\omega(Q)}\right)\right\rangle \quad \text{(S2)}$$

where the upper bar denotes the ensemble average for a given quantity.

Figure S2 shows the variation of δ_d with respect to N_{iso} and A. The upper bound of δ_d is about 30% of the initial resonant frequency (Fig. S2c), which is obtained with smaller N_{iso} and higher A in the lower-order case: the isospectrality for a smaller portion of eigenmodes with the stronger perturbation of optical potentials. Notably, δ_d directly determines the range of achieving the coupling of Q and the decoupling of Q^c. Therefore, the value of δ_d in the valid range of A (see Fig. 4 in the main text) characterizes the performance of selective modal filtering.

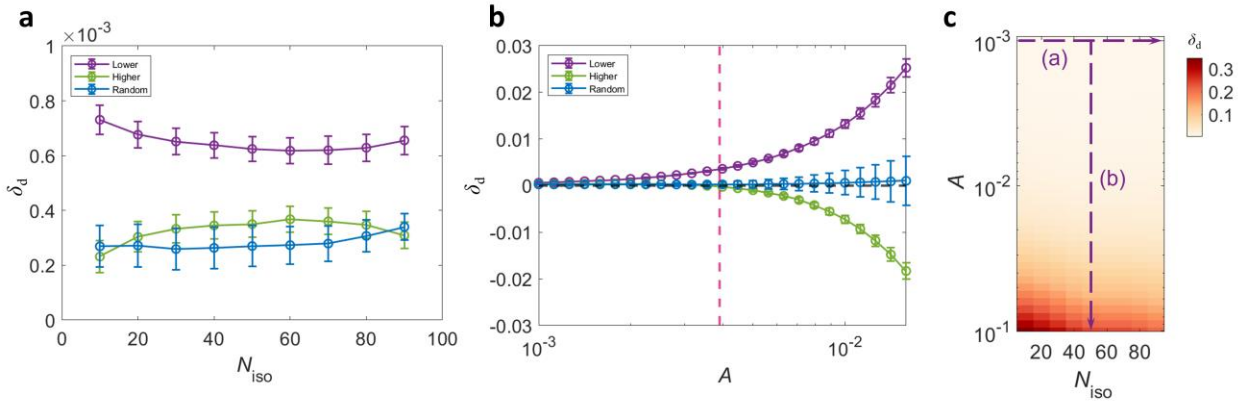

Figure S2. The upper bound analysis of Q^c perturbation. (a,b) Ensemble-averaged δ_d with respect to (a) N_{iso} for $A = 0.001$ and (b) A for $N_{iso} = 50$. The dashed line in (b) represents the upper limit of A for the first-order perturbation validity for the higher-order case, as demonstrated in Fig. 4c in the main text. (c) Ensemble-averaged difference δ_d with respect to (N_{iso}, A) in the lower-order case. The purple dashed lines denote the lower-order cases in (a) and (b). 100 random realizations are examined per ensemble. $L_x = 10$, $L_y = 10$, $M = 100$, $N = 280$, $\omega_L = 1$, $\delta = 0.01$ and $\kappa_0 = 0.01$ for all cases.

Note S3. Efficiency of multimodal filtering

Figure S3 shows the analysis on the efficiency of the multimodal filtering in terms of the spectral broadening with κ and the perturbation strength A. Figure S3a shows that the efficiency η (blue solid line), the ratio between the filtered spectral intensities averaged over the target (Q) and remaining (Q^c) eigenmodes, decreases with increasing κ due to the altered spectral distribution of the original and perturbed lattices, while the coupled intensity to the perturbed lattice (red solid line) increases.

To examine the sideband coupling of Q, we define γ that estimates the sideband coupling noise in the entire decoupling spectral range near Q, as follows:

$$\gamma \equiv \frac{\int_{\min(\omega\in Q)}^{\max(\omega\in Q)} I_{\mathrm{f}}(\omega)d\omega - \sum_{\omega\in Q} I_{\mathrm{f}}(\omega)\delta\omega}{\left[\max(\omega\in Q)-\min(\omega\in Q)\right]\overline{I_{\mathrm{f}}(Q)}}, \quad \text{(S3)}$$

where $I_{\mathrm{f}}(\omega)$ denotes the filtered intensity averaged in the perturbed lattice at ω and $\delta\omega = 2\times10^{-5}\omega_{\mathrm{L}}$ is the spectral discretization for the integration. Notably, a very small value of $\delta\omega$ guarantees highly selective estimation of the Dirac-delta-function-like signal and the sideband noise. Figure S3b shows that a small external coupling coefficient κ guarantees the suppression of the unwanted sideband coupling.

We also measure the averaged η over a set of 100 random inputs with respect to the perturbation strength A (Fig. S3c). In setting A, there is the optimum point due to two different mechanisms; while increasing A allows for the sufficient decoupling of the eigenmodes in Q^c, the value of A that derives the energy shift close to the free spectral range between eigenmodes ($A \sim 10^{-3}$ for our structure) starts to derive the unwanted coupling in Q^c.

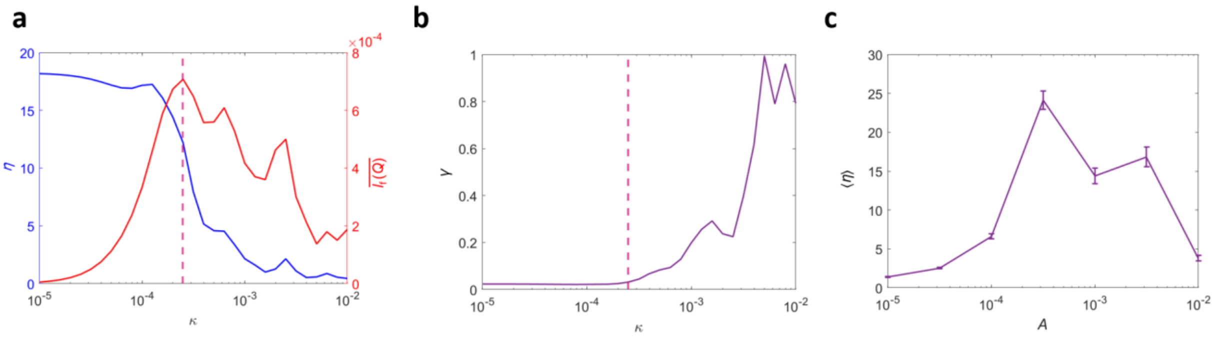

Figure S3. Multimodal filtering efficiency. (a) The efficiency η (blue solid line), and the intensity averaged over Q and the perturbed lattice (red solid line) are shown with respect to κ for a random input example. (b) The noise γ with respect to κ for a single input. The dashed lines in (a) and (b) represent the roughly estimated upper limit of κ determined by the transition behavior of the averaged intensity in (a). (a,b) $A = 0.001$. (c) The averaged η with a set of random inputs for the lower-order mode case with respect to the perturbation strength A. The 100 input realizations are examined per ensemble. $\kappa = 10^{-4}$. (a-c) share the same lattice of Fig. 5a in the main text. The error bar denotes a quarter of the standard deviation for an ensemble. $L_x = 10$, $L_y = 10$, $M = 100$, $N = 280$, $\omega_L = 1$, $\delta = 0.01$, $\kappa_0 = 0.01$ and $N_{iso} = 50$.
